# Effects of Strength Training and Anabolic Steroid in the Peripheral Nerve and Skeletal Muscle Morphology of Aged Rats

**DOI:** 10.3389/fnagi.2017.00205

**Published:** 2017-06-30

**Authors:** Walter Krause Neto, Wellington de A. Silva, Adriano P. Ciena, Ricardo Aparecido Baptista Nucci, Carlos A. Anaruma, Eliane F. Gama

**Affiliations:** ^1^Department of Physical Education, Laboratory of Morphoquantitative Studies and Immunohistochemistry, São Judas Tadeu UniversitySão Paulo, Brazil; ^2^Department of Physical Education, Laboratory of Morphology and Physical Activity, São Paulo State University “Júlio de Mesquita Filho”Rio Claro, Brazil

**Keywords:** exercise, elderly, somatic nerve, male hormone, androgenic hormone

## Abstract

Thirty male 20-month-old *Wistar* rats were divided into groups: IC—initial control (*n* = 6), FC—final control (*n* = 6), AC—anabolic hormone control (*n* = 6), ST—strength trained (*n* = 6) and STA—strength trained with anabolic hormone (*n* = 6). All groups were submitted to adaptation, familiarization and maximum load carrying test (MLCT). Strength training (6–8×/session with loads of 50%–100% MLCT, 3×/week and pause of 120 s) was performed in ladder climbing (LC) for 15 weeks. The administration of testosterone propionate (TP) was performed 2×/week (10 mg/kg) in animals in the AC and STA groups. After the experimental period, animals were euthanized and the tibial nerve and plantaris muscle removed and prepared for electron transmission and histochemistry. To compare the groups we used one-way ANOVA (*post hoc* Bonferroni), student’s *t*-tests for pre vs. post (dependent and independent variables) comparisons and significance level set at *p* ≤ 0.05. The following significant results were found: (a) aging decreased the number of myelinated axon fibers; (b) use of isolated TP increased the diameter of myelinated fibers, along with increased thickness of myelin sheath; (c) ST increased area of myelinated and unmyelinated fibers, together with the myelin sheath. These changes made it possible to increase the area occupied by myelinated fibers keeping their quantity and also reduce the interstitial space; and (d) association of anabolic steroid and ST increased the area of unmyelinated axons and thickness of the myelin sheath. Compared to ST, both strategies have similar results. However, Schwann cells increased significantly only in this strategy.

## Introduction

Several changes in the central (CNS) and peripheral nervous system (PNS) are usually associated with the aging process (Lupien et al., 1998; Tanaka et al., 2005; Burke and Barnes, 2006). In the peripheral nerves, there is a reduction in the quantity of myelinated fibers, loss of the interaction between the axon and Schwann cells, division of the myelin sheath, and redundant changes between the myelin and Ranvier nodes (Mortelliti et al., 1990; Hinman et al., 2006; Wang et al., 2007). Also, the age-related decay of the neuromuscular system results in decreased strength production and contraction velocity, problems in maintaining physical balance, higher risk of accidents and falls and difficulty in performing daily tasks. Collectively, these results may negatively influence the quality and quantity of life of the elderly (Deschenes, 2011).

There is a non-linear relationship between loss of muscle mass and strength causing a decrease in muscle quality (muscle strength per unit of muscle mass; Goodpaster et al., 2006; Delmonico et al., 2009). Changes related to chronological age progression in motoneurons and peripheral nerves appear to play a key role in the decline of motor function (Kanda and Hashizume, 1998). Exercise and physical training are known to have beneficial effects on the PNS (Booth et al., 2002; Wilson and Deschenes, 2005; Deschenes, 2011; Nishimune et al., 2014). Evidence has shown that physical training potentiates the muscular function of aged humans and animals and induces morphological, biochemical and physiological alterations in spinal motoneurons of young and middle-aged animals (Brown et al., 1992; Coggan et al., 1992; Nakano et al., 1997; Kanda and Hashizume, 1998; Shokouhi et al., 2008). Recently, Shokouhi et al. (2008) reported that aerobic exercise protects the peripheral nerves from the action of time by attenuating oxidative reactions and preservation of myelin sheath and Schwann cells from pathological changes that may occur during the process of aging. Nevertheless, data on the effects of strength training on peripheral nerves of the aged are unknown.

In general, significant changes in hormone levels are associated with the aging process and sarcopenia (Kovacheva et al., 2010; Vitale et al., 2016). Thus, administration and/or hormone replacement with androgens also gains space in the field of study of changes related to the advancement of chronological age (Borst and Mulligan, 2007). Androgenic anabolic steroids (AAS) has been studied for the treatment of several conditions, such as severe burns, cachexia of individuals with aids and cancer, severe anemia, osteoporosis, frailty syndrome, among others (Basaria et al., 2001; Kicman, 2008). During the last decade, many publications have demonstrated a significant dose-dependent increase in strength and muscle mass, myofibers cross-sectional area (CSA) enlargement, myonuclei and satellite cells aggregation in the muscles of the elderly (Bhasin et al., 2005; Sinha-Hikim et al., 2006; Sattler et al., 2011). A recent meta-analysis has shown that the effects of AAS on the lean mass of the elderly are dose-dependent and time-consuming (Krause Neto et al., 2015). In general, several reviews have discussed the effects of these steroids on the CNS, since the first observations were reported in this tissue (Melcangi et al., 2011; Melcangi and Panzica, 2014). However, more recent studies have indicated that PNS also synthesizes and metabolizes steroids and can be a target for these molecules. In fact, neuroactive steroids exert important physiological functions in the PNS and act on the glial and neuronal compartments (Rodriguez-Waitkus et al., 2003; Melcangi et al., 2005, 2011). Thus, it is possible that AAS can also modulate adjustments in the PNS.

This study aimed to analyze the effects of strength training associated with the administration of exogenous testosterone in the peripheral nerve and skeletal muscle morphology of aged *Wistar* rats.

## Materials and Methods

This study was authorized (Committee on Ethics in Animal Use—Protocol 001/2013) and carried out at the Laboratory of Morphoquantitative Studies and Immunohistochemistry of the São Judas Tadeu University (LEMI-USJT). Logistical and technical support were given by the Laboratories of Morphology and Physical Activity of the Institute of Biosciences of the São Paulo State University “Júlio de Mesquita Filho” (UNESP-Rio Claro) and Electronic Microscopy of the Institute of Biomedical Sciences of the University of São Paulo (ICB-USP).

### Division of Animals

Thirty male rats (*Rattus norvegicus*) of the *Wistar* line, aged 20 months, from the São Judas Tadeu University were divided into five groups using the randomized method. Each group was submitted to a type of procedure, as described below: initial control (IC)—sedentary animals that were used as IC of the procedures (*n* = 6); final control (FC)—sedentary animals which remained as FC of the procedures (*n* = 6); AC—sedentary animals which received injections of anabolic steroid (*n* = 6); strength trained (ST)—animals which underwent strength training (*n* = 6) and STA—animals which underwent strength training and received injections of anabolic steroid (*n* = 6). Rodents were housed in polypropylene boxes (a maximum of three animals each) kept in controlled environmental conditions of temperature (22°C) and illumination (12-h light and 12-h dark). For all groups, we provide commercial reference food for rats and water *ad libitum*.

### Strength Training

Training protocol was performed during the active period of the animal (nocturnal). For this, we adapted the animal’s accommodation and training room by reversing the light-dark cycle (lights programmed to remain off from 7 o’clock in the morning to 7 o’clock in the evening). The strength training sessions were done between 11 a.m. and 4 p.m. of the scheduled days, using the ladder climbing (LC) equipment (110 cm of height, 80° of inclination and distance of 2 cm between each step).

Rats were conducted to adaptation process during five sessions on consecutive days (Monday to Friday) without any additional burden attached to the animal’s tail. In the next week, maximum loaded carrying capacity tests (MLCT) were applied using two familiarization sessions. During testing sessions, animals should climb the ladder as many times as possible from a given initial overload (50% BWa), adding to each successful attempt a new percentage. Testing session was conducted until the animal was unable to climb the ladder for at least two consecutive trials (failure). Between each attempt, the animal had an interval of 2 min.

From the week following the completion of the MLCT, rodents were conducted to strength training protocol during 15 weeks (45 sessions). In the first session, rats climbed the ladder twice in each of the following workloads: 50, 75 and 100% of the final load of the MLCT. If the animal could climb the ladder twice to 100%, an extra 30 g was added on the last climbing load. The animal, then, was able to climb the ladder two more times, totaling at the end eight maximum climbs. In the subsequent sessions, was calculated the same percentages (50, 75 and 100%) from the new maximum load reached in the previous session (Harris et al., 2010). If the animal could not complete the protocol with eight maximum climbs, the same loads were maintained for the next training session. Each rodent had a 2-min interval between each climb. Training was performed three times per week on alternate days.

### Anabolic Steroid

The PERINON® anabolic steroid from the Perini laboratory (100 ml vial containing 200 mg/20 ml of testosterone propionate [TP]) was used in this study (10 mg/kg BWa/week). Animals were weighted every Tuesday to recalculate the application dosage. TP administration was done by intraperitoneal injection, twice a week (Tuesdays and Fridays), starting from the first day of the experiment, in the AC and STA groups. The duration of treatment was concomitant with the strength training period of the ST and STA groups (i.e., 15 weeks).

### Euthanasia, Collection and Preparation of the Material for Analysis

The animals were euthanized using the CO_2_ inhalation method, according to the schedule of each experimental group. After euthanasia, tibial nerve and plantaris (PL) muscle were removed and prepared for electronic transmission and histochemistry analysis. Tibial nerve was chosen because of its innervation in the predominant muscles of the posterior region of the lower limbs. The PL muscles were chosen according to their predominance of muscle fiber typology and action during the climbing exercise. The PL is glycolytic and predominantly formed of faster contraction myofibers (type II). Thus, an incision was made in the posterior portion of the right knee of the animal up to his ankle to expose the tibial nerve and PL muscle. After the tibial nerve and PL muscles are exposed, cleaned of fat and connective tissue, we quickly prepared them for specific analyses of light and electronic transmission microscopy.

### Electronic Transmission Microscopy

After incision in the posterior portion of the right knee, we removed a fragment of approximately 0.5 cm in length from the tibial nerve. Nerve fragment was placed in 2.5% glutaraldehyde-fixing solution in phosphate buffer (0.2 M, pH 7.3) for 3 h. Then the material was washed three times with the same buffer solution for 5 min at a time. Subsequently, it was placed in a solution of 1% osmium tetroxide in phosphate buffer for 2 h. The fragments remained overnight in 0.5% uranyl acetate. In the morning, it was washed with the plug, dehydrated in increasing series of alcohol and propylene oxide for 8 h, under rotation. The nerves were included in the pure resin (Spurr), in the position that the nerve fibers were cross-sectioned. The material remained in this step for 5 h and then left in the same resin at 60°C for a further 3 days. After completion of the material preparation procedure, the histological slides were prepared and the tissue stained with toluidine blue. After selection of the fields in the semifinished sections, the ultra-thin sections were obtained with a diamond knife, in ultramicrotome (Sorvall MT-2), and contrasted with uranyl acetate and lead citrate, finally being analyzed by the electronic transmission microscope.

The material was taken to the electronic transmission microscope (Jeol JSM1010, ICB, USP) and photomicrographs of the nerves were captured in cross-shaped fields starting from the top to the bottom of the image and from left to right. In this way, we were able to photograph the material trying to capture the nerve as a whole. Photomicrographs were done using a magnification of 1500 and 3000 times for stereological and morphometric analysis, respectively. All final preparation of the material and images was done by the Department of Anatomy and Laboratory of Electronic Microscopy of ICB-USP.

For the stereological study, we captured 25 images of each group in the transmission electron microscope with a final magnification of 1500 times. The following variables were analyzed and quantified:

- Density of volume (Vv): myelinated fibers (Vv [MF]), unmyelinic axons (Vv [UA]) and interstitium (Vv [inters]). A system with 588 points was placed on each frame, covering 100% of the content contained in the image. The points that fell on each of the previously mentioned structures were quantified, and the data were automatically transformed into percentages relative to the total number of points.
- Numerical density (Nv): myelinated fibers (Nv [MF]), unmyelinated axons (Nv [UA]) and Schwann cells at the nuclei level (Nv [SC]). The cited structures, contained in each frame, were quantified, excluding those that touched the lower and left edges of the image. We also calculated the ratio of unmyelinated axons by myelinated fibers (UA/MF), through the quotient between the number of unmyelinated axons and myelinated fibers. In order to measure and evaluate these parameters, we used the software Image J.

For the morphometric study, we captured 35 images of each group using a 3000-fold increase and performed the following measurements: CSA (μm^2^) of myelinated fibers, myelinated and unmyelinated axons, mean diameter (μm) of myelinated fibers and axons, thickness (μm) of the myelin sheath and G ratio. To measure the CSAs, each structure of interest in the image was surrounded. Subsequently, the mean diameters were calculated from the mean between largest and smallest diameters of each structure. To calculate the G ratio, we used the quotient between the diameter of the axon and the myelinated fiber. Finally, the mean thickness of the myelin sheath was determined by the average of four equivalent cross-shaped traces on the image. For this, we use the Axiovision 4.8 program.

### Histochemistry

The PL muscle was embedded in liquid nitrogen and kept at −80°C until analysis. The assembly and preparation of the slides were done at the Laboratory of Morphology and Physical Activity of UNESP-Rio Claro.

For staining with Nicotinamide adenine dinucleotide tetrazolium reductase (NADH-TR), we cut the tissue in a cryostat −20°C (thickness of 8 μm), incubate the slides for 40 min at a temperature of 37°C in solution containing 8 mg of NADH (reduced), 10 mg of NBT and 0.2 M Tris buffer (pH 7.4). Next, we washed the material in 30%, 60% and 90% increasing acetone P.A then return to 60% and 30%. Finally, we mounted on Jeli glycerin.

For morphometry, we analyzed 35 photographs of each group with a final magnification of 400×. Measurements were made in software Axiovision version 4.8 coupled to the light microscope (Zeiss). In each slide, it was possible to measure the cross-sectional area (μm^2^) of oxidative muscle fibers type I (dark blue) and glycolytic myofibers type II (white).

### Statistical Analysis

All data are presented by mean and standard deviation (SD). For statistical comparison between the different groups, one-way ANOVA (*post hoc* Bonferroni) was used. For comparison of the PRE vs. POST results of the same group Student’s *t*-test was calculated for dependent samples. To compare the same parameters among the trained groups, we used a *t*-test for independent samples. For the statistical calculations, we used SPSS software version 21.0 and set the level of significance at *p* ≤ 0.05.

## Results

### Training Load

**Figure 1:**
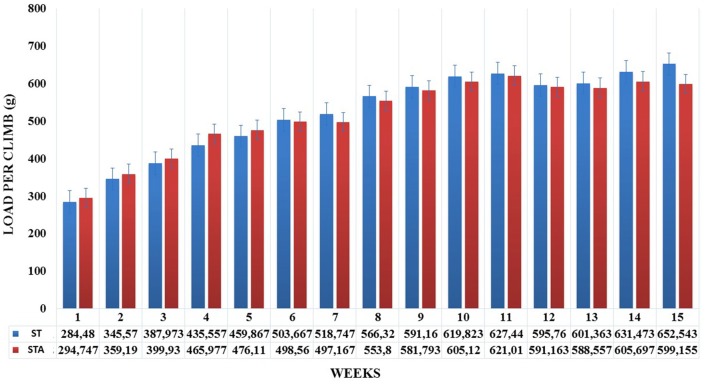
Load per climb (g) during 15 weeks of experimentation from groups strength trained (ST) and strength trained plus anabolic steroid (STA).

Groups ST and STA increased significantly load per climb (*p* < 0.05), however, neither difference was found between groups (Figure 1).

**Figure 2:**
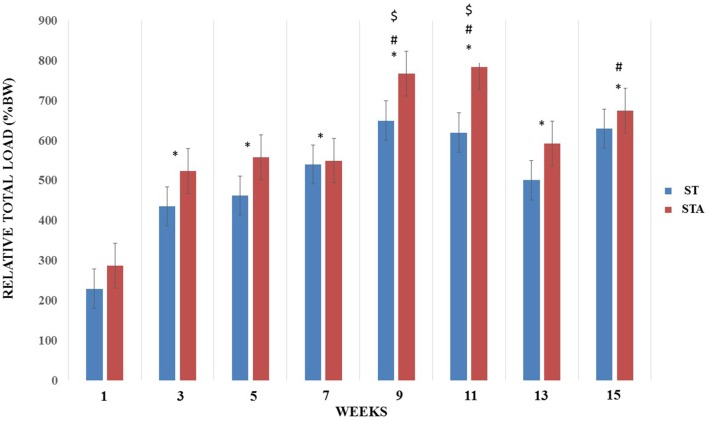
Relative total load (%BW) progression during 15 weeks of strength training from groups trained (ST) and trained plus anabolic steroid (STA). *Significantly different from week 1; ^#^significant different from week 3; ^$^significantly different from week 5.

Relative load increased significantly from week 3–15 compared to week 1 (*p* < 0.05). Both groups presented higher training loads at weeks 9, 11 and 15 compared to week 3 (*p* < 0.05). Also, relative loads were higher at weeks 9 and 11 compared to week 5 (*p* < 0.05; Figure 2).

**Figure 3:**
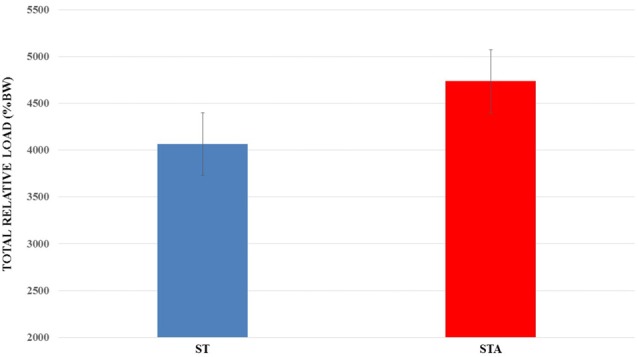
Total relative load (%BW) trained during 15 weeks of experimentation from groups trained (ST) and trained plus anabolic steroid (STA).

Total relative load is presented in Figure 3. There was no statistical difference between groups (*p* > 0.05).

**Figure 4:**
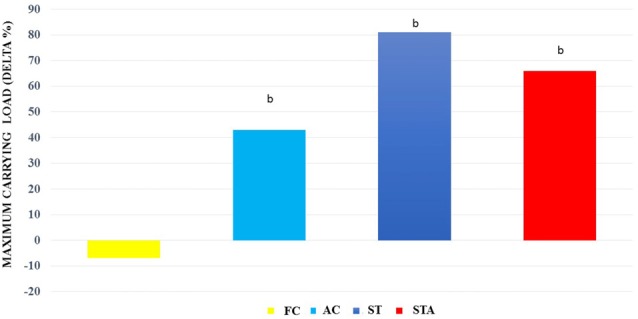
Maximum carrying capacity loads (Delta %) from *Wistar* rats of groups aged control (final control, FC), anabolic steroid control (AC), ST and strength trained plus anabolic steroid (STA). ^b^Significantly different from group FC (*p* < 0.05).

For maximum carrying load testing, groups AC, ST and STA presented higher loads than FC (*p* < 0.05; Figure 4).

### Tibial Nerve

Data are presented in Table 1. The FC group presented greater CSA of unmyelinated fibers than IC (*p* < 0.05). Nv[MF] was lower in FC than IC (*p* < 0.05). For other analyses, we did not find any significant differences.

**Table 1:** Tibial nerve morphometry of *Wistar* rats after 15 weeks of experimentation in the early aged (initial control, IC), final (final control, FC), testosterone (anabolic control, AC), trained (strength trained, ST) and testosterone+trained (strength trained anabolic, STA) groups.

	Groups						
Variables	IC	FC	AC	ST	STA	*F*	*P* value
	**Cross-section area (μm^2^)**						

Myelinated fiber	2.32 ± 1.86	3.05 ± 2.01	3.85 ± 3.18^a^	3.42 ± 2.57^a^	2.76 ± 1.78^c^	12.065	0.000
Myelinated axon	0.94 ± 0.77	1.17 ± 0.87	1.25 ± 1.05^a^	1.16 ± 0.92	0.89 ± 0.75^c^	6.597	0.000
Unmyelinted axon	0.026 ± 0.016	0.038 ± 0.023^a^	0.031 ± 0.023	0.037 ± 0.026^a^	0.042 ± 0.024^a,c^	8.859	0.000
	**Diameter (μm)**						

Myelinated fiber	1.84 ± 0.54	2.11 ± 0.44	2.87 ± 0.81^a,b^	2.33 ± 0.37	1.91 ± 0.21^c^	7.503	0.000
Myelinated axon	1.26 ± 0.34	1.36 ± 0.32	1.61 ± 0.3	1.35 ± 0.14	1.17 ± 0.27^c^	4.370	0.002
G ratio	0.68 ± 0.04	0.64 ± 0.03	0.62 ± 0.3	0.59 ± 0.1	0.61 ± 0.15	0.513	0.765
	**Thickness (μm)**						

Myelin sheat	0.31 ± 0.07	0.35 ± 0.06	0.47 ± 0.12^a.b^	0.44 ± 0.07^a,b^	0.45 ± 0.06^a.b^	17.200	0.000

*Values are presented as mean ± standard deviation. G ratio = axon diameter/myelinated fiber. ^a^Indicates a significant difference in relation to IC (p ≤ 0.05). ^b^Indicates a significant difference in relation to FC (p ≤ 0.05). ^c^Indicates significant difference in relation to AC (p ≤ 0.05)*.

Testosterone therapy promoted a significant increase in the CSA of myelinated fibers and axons (*p* < 0.05), as well as the mean diameter of the myelinated fibers compared to the IC group (*p* < 0.05). As for the thickness of the myelin sheath, the AC group showed a significant increase in comparison to IC and FC (*p* < 0.05). However, testosterone administration was not effective in avoiding the reduction of Nv[MF] (*p* < 0.05).

**Figure 5:**
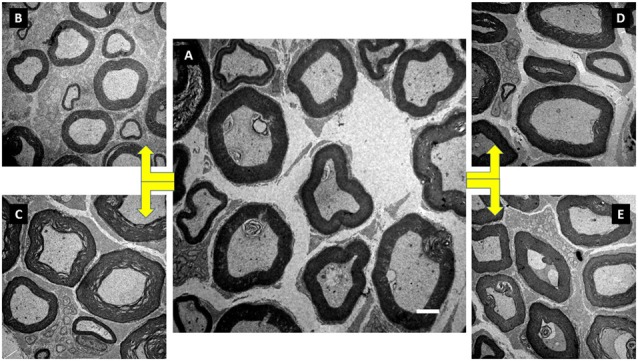
Illustration of the tibial nerve morphology from *Wistar* rats of groups initial control (IC; **A**), FC **(B)**, anabolic steroid control **(C)**, ST **(D)** and strength trained plus anabolic steroid **(E)**. Electronic microscopy. Magnification of 3000×.

The ST group increased the CSA of myelinated fibers and unmyelinated axons (*p* < 0.05). In contrast to IC and FC, strength training significantly increased myelin sheath thickness (*p* < 0.05). Regarding Vv[MF], the ST group showed a larger volume of occupied area compared to FC and AC (*p* < 0.05; Figure 5). Inversely proportional was shown in Vv[INTERS], whose ST group had lower occupied area compared to FC and AC (*p* < 0.05; Table 2).

**Table 2:** Tibial nerve stereology of *Wistar* rats after 15 weeks of experimentation in the early aged (IC), final (FC), testosterone (AC), trained (ST) and testosterone+trained (STA) groups.

	Groups						
Variables	IC	FC	AC	ST	STA	*F*	*P* value
	**Vv**						

Myelinated fiber	65.87 ± 8.24	59.05 ± 4.87	60.3 ± 8.42	70.83 ± 5.32^b,c^	66.32 ± 5.51	4.615	0.001
Unmyelinated axon	3.78 ± 2.3	1.98 ± 1.67	4.13 ± 2.35	4.85 ± 3.8	3.75 ± 2.83	1.607	0.174
Intersticium	30.36 ± 7.68	38.97 ± 4.95	35.36 ± 7.98	24.32 ± 6.73^b,c^	29.93 ± 6.99	5.690	0.000
	**Nv**						
Myelinated fiber	11.07 ± 4.17	7.73 ± 3.26^a^	7.13 ± 2.85^a^	9.33 ± 2.47	9.6 ± 2.03	5.447	0.000
Unmyelinated axon	19.53 ± 18.42	12.2 ± 9.85	17.2 ± 16.96	24.13 ± 18.84	24.13 ± 17.9	1.118	0.326
Schwann cell	0.67 ± 0.87	0.3 ± 0.67	0.3 ± 0.48	0.2 ± 0.42	1.33 ± 0.78^b,c,d^	4.766	0.001
UA/MF ratio	1.5 ± 1.2	1.67 ± 1.48	2.23 ± 1.55	2.45 ± 1.41	2.63 ± 2.03	1.701	0.143

*Values are presented as mean ± standard deviation. Vv, volume density (%). Nv, numerical density (number). UA/MF ratio, unmyelinated axon/myelinated fiber. ^a^Indicates a significant difference in relation to IC (p ≤ 0.05). ^b^Indicates a significant difference in relation to FC (p ≤ 0.05). ^c^Indicates significant difference in relation to AC (p ≤ 0.05). ^d^Indicates a significant difference in relation to ST (p ≤ 0.05)*.

The combination of strength training with TP significantly increased the area of unmyelinated axons compared to the IC and AC group (*p* < 0.05). The STA group showed areas of the transverse section and mean diameter of MF and MA statistically lower than AC (*p* < 0.05). However, the thickness of the myelin sheath was statistically higher than IC and FC (*p* < 0.05). For stereology, the STA group showed higher Schwann cell number than FC, AC and ST (*p* < 0.05).

### Plantaris Muscle

**Figure 6:**
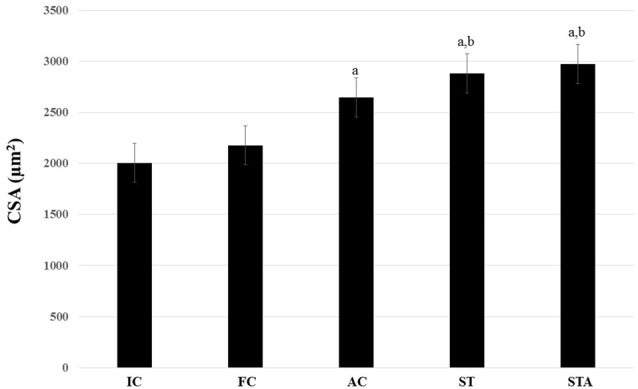
Cross-sectional area (CSA) of the oxidative type I myofibers from the plantaris muscle of *Wistar* rats after 15 weeks of experimentation in the early aged (IC), final (FC), testosterone (AC), trained (ST) and testosterone+trained (STA) groups. ^a^Indicates significant difference from IC (*p* ≤ 0.05); ^b^Indicates significant difference from FC (*p* ≤ 0.05).

The AC group significantly hypertrophied type I myofibers compared to IC (*p* < 0.05). ST and STA groups demonstrated significant larger type I myofibers compared to IC and FC (*p* < 0.05; Figure 6).

**Figure 7:**
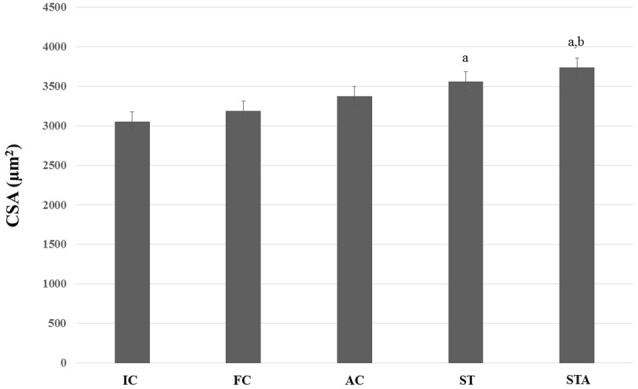
CSA of the glycolytic type II myofibers from plantaris muscle of *Wistar* rats after 15 weeks of experimentation in the early aged (IC), final (FC), testosterone (AC), trained (ST) and testosterone+trained (STA) groups. ^a^Indicates significant difference from IC (*p* ≤ 0.05); ^b^Indicates significant difference from FC (*p* ≤ 0.05).

As for type II myofibers, both groups showed larger fibers than IC (*p* < 0.05). However, only STA group had significant results compared to FC (*p* < 0.05; Figure 7).

## Discussion

Clearly, it is mentioned in the literature that the advance of age affects the function and regeneration of peripheral nerves in both human and experimental models (Jeronimo et al., 2008; Scheib and Höke, 2016). Ugrenović et al. (2016) suggest that a better understanding of peripheral nerve changes provided by aging may enhance the interpretation of their pathological changes as well as their understanding of their regeneration at various ages. In this study, we did not observe any changes in most of the variables between groups IC and FC. However, the literature shows a great variability of evidence, citing variations between the types and functions of peripheral nerves, nerve portion, species and rodent lineages analyzed. Jeronimo et al. (2008) studied the sural nerve of *Wistar* rats evaluating the proximal and distal portions of both right and left limbs. The authors found no statistical difference in the MF area between the ages of 360, 640 and 720 days. However, the myelinated axon area was significantly reduced in the distal segment of the older sample. The explanation may be the effect of aging on the structure of the motoneurons since we know that the number of motoneurons decreases with age (Kanda and Hashizume, 1998). Nevertheless, there is no clue as to when this process begins or how the process occurs. Hashizume and Kanda (1995) evaluated motor neurons of Fisher 344 rats and found a reduction in the number of motor nuclei of the medial gastrocnemius muscle nerve at the age of 27 months. The authors explained that this may be due to cell death or an increase in the number of unlabeled nuclei. If the axon no longer extends, the axoplasmic transport will not occur, but despite this, such demonstration did not occur to the ulnar nerve. Furthermore, changes in other components of the PNS, such as a neuromuscular junction, may precede peripheral nerve changes (Deschenes et al., 2010). This fact brings to light the different effect of aging on the different nerves and their morphology. Recently, Sakita et al. (2016) demonstrated significant atrophy of the perimeter of myelinated axons in rats between 20 weeks and 90 weeks of age. Ceballos et al. (1999) studied the tibial nerve of mice between 6 months and 33 months of age, describing the variability of effects on different MF sizes. The authors demonstrated significant atrophy of nerve fibers only in the older groups (27 + 33 months) compared to the 6-month group. Perhaps, reduction on MF numerical density may precede it. Thus, we can presume that atrophy of these fibers should occur later than expected.

The myelin sheath is a fundamental structure in the healthy functioning of healthy nerves (Magnaghi et al., 2001; Melcangi et al., 2003). In the present study, we did not find any variation in the thickness of the myelin sheath between IC and FC groups. Our data are corroborated by several studies in the literature. Shen et al. (2011) investigated morphological and functional changes in the sciatic nerve during maturation and aging processes. The authors mentioned a gradual increase of myelin sheath lamellae from 1 week of life to 12 months of age. From this point up to 18 months, the myelin sheath remained unchanged. Electrophysiological analysis showed that the amplitude of muscular action potentials gradually increased from 1 week to 18 months of age, showing a positive linear correlation with the number of lamellae in the myelin sheath. Other studies, whose focus was to evaluate more advanced ages, also showed no changes in the thickness or perimeter of the myelin sheath (Hashizume and Kanda, 1995; Jeronimo et al., 2008). However, Ceballos et al. (1999) found a significant variation in myelin sheath thickness, which was demonstrated between the ages of 16 months and 22 months of the tibial nerve, without any subsequent change in the G ratio. Knowing that the portion of the nerve analyzed can influence the results investigated, Sakita et al. (2016) demonstrated that both myelin sheath thickness and perimeter of the distal portions of nerves of *Wistar* rats at 90 weeks of age were significantly lower than the younger group. Thus, it is evidenced that aging may initially affect the distal portion of peripheral nerves before proceeding towards the soma of the motoneuron.

The ratio G corresponds to the myelinization index of MAs, being an important variable in the aging process. According to Rushton (1951) values of G ratio between 0.6 and 0.7 would be the best for a good and maximum speed of conduction in MF. Our data were corroborated by Sakita et al. (2016) who also demonstrated maintenance of the G ratios of the tibial nerve of rats between 20 weeks and 90 weeks of age. Nakayama et al. (1998) reported that there was no reduction in the rate of conduction of action potentials in the MF of old rats. Despite this, Jeronimo et al. (2008) found a significant reduction of the G ratio at the age of 720 days. It is noteworthy that in all the studies cited, different nerves were analyzed.

Interesting variations in the peripheral neuronal structure of aged samples can be found in the literature (Ceballos et al., 1999). It has been reported that MF suffers more from the aging process than unmyelinated fibers (Sato et al., 1985; Hinman et al., 2006; Shen et al., 2011; Sakita et al., 2016). This fact is explained by the great variability of myelinic alterations found along the advancing age (Ceballos et al., 1999; Ugrenović et al., 2016). Here, the CSA of the unmyelinated axons in FC was 46.2% greater than IC. Ceballos et al. (1999) reported a significant reduction of 50% of the UA area with advancing age. The authors suggest that part of their results, and also in our case, it was difficult to discriminate the original unmyelinated axons of the axons resulting from the degeneration and regeneration process. Thus, such results may be overestimated. Sato et al. (1985) demonstrated that while there is a reduction in the electrical impulse conduction velocity of myelinated fibers of the peripheral nerve of the gastrocnemius and soleus muscles, the same was not found for unmyelinated fibers. The reason for this may lie in the fact that the major changes induced by aging occur in the quality and morphology of the myelin sheath.

In the stereological study, Nv[MF] analysis showed that the FC group presented 30.2% less myelinated fibers than IC. Ceballos et al. (1999) demonstrated similar results in both analyses (Vv[MF and UA] or Nv[MF and UA]). However, significant reductions of these parameters have only been demonstrated in ages from 27 months. Such a relationship is probably compensatory, where the MF area increases to compensate the reduction in its number.

According to Tauchi et al. (1971), glycolytic fibers decreased in size while oxidative fibers decreased in number. Here, no change was observed in *plantaris* muscle from IC and FC. Our data are corroborated by Deschenes et al. (2015) that demonstrated an increase in the oxidative myofibers proportion and enlargement of glycolytic ones, without any change in the CSAs. Yet, Deschenes et al. (2016) showed reduced type I and IIa CSA, with an increase in the proportion of type IIx myofibers.

The administration of steroid hormones in the treatment of peripheral trauma is becoming increasingly important (Mitro et al., 2014; Giatti et al., 2015; Sarabia-Estrada et al., 2015). However, it is not known whether this practice is consistent in disrupting or reversing the neuronal loss observed over the course of aging. Yet, a likely compensatory effect, similar to that seen in the aging process in Nv[MF] variable, occurred in both area and diameter of MFs with TP administration. In order to understand what happened, we sought evidence of the form of action and the metabolism of testosterone in the peripheral nerve. Melcangi et al. (1990) indicated that 5α-reductase, an enzyme that converts testosterone to its “active” metabolite dihydrotestosterone (DHT), is highly concentrated in a White matter of the CNS, composed mainly of myelinated fibers. The authors conducted the first study in order to evaluate the possible presence of this enzyme activity in myelinated peripheral nerves. For this purpose, 5α-reductase activity was measured in the sciatic nerve of rodents and compared to that present in the cerebral cortex and subcortical white matter. The study was conducted using a group of healthy adult male rats (60–90 days old) and an aged rodent sample (20 months old). The data obtained in the adult animals indicated the presence of an active metabolism of testosterone at the sciatic nerve level. In this structure, testosterone is actively transformed into DHT and 3α-diol, with DHT formation being equal to that found in subcortical white matter and higher than found in the cerebral cortex. Furthermore, in disagreement with what occurs in CNS structures, where 3α-diol is produced only in small amounts, in the sciatic nerve this metabolite is produced in amounts similar to those of DHT. On the other hand, the study of the aged group demonstrated that in the sciatic nerve, the formation of DHT and particularly 3α-diol are much lower than in younger animals. So, not only can testosterone (T) decrease with advancing age, but also your local metabolism. Pannérec et al. (2016) provided consistent results citing that susceptibility to sarcopenia is closely linked to a neuromuscular decline in rats and humans, and even a dysregulation of steroid metabolism in the PNS is an early event in this process. In this way, treatment with T or its metabolites can interrupt or delay deleterious effects, whether traumatic or related to aging. Cai et al. (2017) examined whether the protective effects of testosterone could be mediated through their androgenic or estrogenic metabolites. Motor neurons that innervate the vastus lateralis muscle of adult rats were selectively deleted. After 4 weeks, the motoneurons that innervate the vastus lateralis muscle were marked and the dendritic ramifications reconstructed in three dimensions. Compared with normal intact animals, partial motor neuron reduction resulted in decreased dendritic length in the remaining motor neurons. Dendritic atrophy was attenuated with both DHT and estradiol to a degree similar to that observed with testosterone, and the attenuation of atrophy was prevented by receptor blockade. Together, these results suggest that the neuroprotective effects on motor neurons can be mediated by steroid hormones and their respective receptors. These findings seem to be of extreme importance, but the action of these neuroprotective effects may be reduced with advancing age (Melcangi et al., 2003).

Among the physiological effects of neuroactive steroids on the PNS, the myelination process was investigated extensively. For example, a major myelin protein, such as the zero glycoprotein (P0), is a target for the action of progestins and their derivatives (dihydroprogesterone [DHP] and tetrahydroprogesterone [THP]) as well as T-derivatives (DHT and 3α-Diol; Magnaghi et al., 1999, 2004; Melcangi et al., 1999, 2005). Furthermore, the peripheral myelin protein 22 (PMP22) is under the control of THP and 3α-diol (Magnaghi et al., 1999, 2004; Melcangi et al., 1999, 2005). These physiological effects are mediated by the activation of classical or non-classical steroid receptors. Observations to date indicate that the expression of P0 is under the control of classical steroid receptors, such as progestin (PR) and AR receptors, whereas that of PMP22, is under the control of a non-classical receptor such as GABA-A receptor (Melcangi et al., 2005). The decrease in the synthesis of P0 and PMP22 and its association with morphological changes in peripheral nerves have been reported during aging (Azcoitia et al., 2003; Melcangi et al., 2003). The effects seen in the treatment with progestins and their derivatives seem to be a peculiarity of this class of neuroactive steroids because neither T nor its derivatives seem to be able to influence the morphological parameters analyzed in these experiments (Azcoitia et al., 2003). Azcoitia et al. (2003) evaluated the effect of P, DHP, THP, T, DHT and 3α-diol on the morphological changes of myelinated fibers of the sciatic nerve of male rats aged 22–24 months. The sciatic nerves of untreated old rats showed general disorganization and a significant reduction in the density of myelinated fibers compared to the nerves of male rats at 3 months of age. In addition, the sciatic nerves of old rats showed a significant increase in the number of fibers with myelin invaginations in the axoplasm and with irregular shapes. Treatments of old mice with P, DHP and THP resulted in a significant increase in the number of myelinated fibers, a significant reduction in the frequency of myelin sheath abnormalities, and a significant increase in the G ratio of small myelinated fibers. Treatments with T, DHT or 3α-diol did not significantly affect any of the morphological parameters examined. A probable explanation for the lack of effects of T on the parameters analyzed rests on the fact that the authors used subcutaneous injections of pure T, whose degradation is faster, and the application time was only 32 days. A recent meta-analysis published by our group demonstrated that treatment time, application form and steroid class may affect the outcomes analyzed (Krause Neto et al., 2015). Further, in our study, thicker myelin sheaths were found, bringing to light the fact that anabolic steroids can somehow stimulate T metabolism and their respective actions on peripheral nerves.

Data about muscle hypertrophy in older samples submitted to testosterone therapy demonstrated an increase in the number of myonuclei (Sinha-Hikim et al., 2002). Kovacheva et al. (2010) found that both types of myofibers showed significant enlargement. According to Katznelson et al. (2006), higher doses of AAS may have a better effect than therapeutic doses for increasing muscle hypertrophy.

The effects of aerobic physical training on non-traumatized and young peripheral nerves include changes in the mean size of myelinated fibers and axons and myelin sheath (Roy et al., 1983). Nevertheless, other studies have failed to demonstrate any change induced by the regular practice of this strategy (Key et al., 1984; Malysz et al., 2010). Key et al. (1984) suggest that exercise intensity and type of training may be critical parameters responsible for the discrepancies seen in the literature. Carbone et al. (2017) demonstrated that resistance training applied to young *Wistar* rats at 75% of body weight load significantly altered morphological parameters of the radial nerve, which were not found in the group that trained with lower intensity (50% BW). To our knowledge, this is the first study that investigated the effects of strength training on the peripheral nerve of old rats.

Our results are interesting, since there was a significant hypertrophy of the MF of the ST group, mainly due to the increase in the thickness of the myelin sheath. Studies with strength training conducted using healthy aged sample are scarce. Thus, we will use as comparison studies with endurance training. Samorajski and Rolsten (1975) evaluated the tibial nerve of C57BL/10 young rodents for 24 months. The animals exercised on a treadmill for 2 h every day. At the end of the training period, the authors demonstrated significant hypertrophy of the nerve fibers. Kanda and Hashizume (1998) examined the number and size of myelinated motor neurons and nerve fibers that innervate the medial gastrocnemius muscle of rats undergoing 10 months of swimming training from 17 months to 27 months of age. The size of the motoneuronal sum was significantly greater than the sedentary group. In addition, the morphological parameters of the peripheral nerve were intermediate to the middle-aged and sedentary group. The authors concluded that long-term training may delay the progressive changes seen during aging. Some mechanisms may be involved in the action of physical training on the effects of time on the peripheral nerve. Shokouhi et al. (2008) studied the effects of long-term training on lipid peroxidation, apoptosis of Schwann cells and ultrastructural changes of the rat sciatic nerve. Three groups of 12-week-old rats ran for 6, 9 and 12 months on a treadmill following an exercise program with a velocity of 22 m/min (7° inclines), 60 min/day and 6 days/week. The results showed that aging was related to an increase in the level of lipid peroxidation in the nerve and a higher number of SC apoptosis in the sedentary group. This same group also showed irregular nerve fibers, with thin myelin sheath and areas of myelin detachment from the axons. In contrast, the trained group had significantly decreased the lipid peroxidation of the nerves and apoptosis of Schwann cells. In the training group, the nerve fibers had a thick myelin sheath with normal folds. These findings suggest that aerobic physical training protected peripheral nerves by attenuating oxidative reactions, preserving Schwann cells and the myelin sheath from pathological changes, which normally occur during aging. Cassilhas et al. (2012) evaluated the effects of resistance training on spatial memory and signaling pathways of brain-derived neurotrophic factor (BDNF) and Insulin-like growth factor-1 (IGF-1), comparing these effects with those of endurance training. After 8 weeks of experimentation, both endurance and strength training showed improvement in learning and memory, in a very similar way. However, both groups had different signaling pathways. Although endurance group showed increased levels of IGF-1, BDNF, TrkB and β-calcium dependent kinase II/calmodulin (β-CaMKII) in the hippocampus, the resistance group showed an induction of peripheral and hippocampal IGF-1 with concomitant receptor activation of IGF-1 (IGF-1R) and Akt in the hippocampus. These distinct pathways culminated in increased expression of synapsin 1 and synaptophysin in both groups. These results demonstrated that both endurance and strength training may employ divergent molecular mechanisms, but achieve similar results on learning and spatial memory. In particular, BDNF seems to be a central modulator of central and possibly peripheral neuroplasticity (Knaepen et al., 2010; Huang et al., 2014). Thus, some studies sought to know whether physical training could also modulate BDNF expression in peripheral cells. Wilhelm et al. (2012) demonstrated that treadmill endurance training potentiates the expression of BDNF in Schwann cells, allowing for a more rapid recovery of traumatized nerves. Yarrow et al. (2010) studied the effect of 5 weeks of strength training on BDNF expression, comparing serum BDNF levels before and after training. Thus, strength exercise induced a robust increase in circulating BDNF and the progressive exercise potentiated this response. Despite this, a recent meta-analysis has shown that endurance exercise is more significant than strength exercise in increasing circulating BDNF levels (Dinoff et al., 2016). This fact can be explained by the intensities and duration of training, besides the age of the sample. According to Forti et al. (2015), it may be necessary that the prescription is made by intensity close to maximum voluntary fatigue and higher values of total volume of load. Thus, further studies are needed to verify whether ST can increase the gene expression of neurotrophic factors in peripheral nerve cells.

Strength training has been considered very effective in decreasing rates of muscle loss and strength of the elderly (Lynch et al., 2007). Wang et al. (2017) demonstrated that heavy strength training increases maximal strength, the rate of force development, work efficiency and muscular hypertrophy, especially among myofibers type II. Corroborating, Conlon et al. (2017) also showed increases in muscular hypertrophy and strength, independently of periodization strategies. Here, ST was effective to induce muscle hypertrophy and strength gains.

Clearly, steroid hormones can modulate the morphological response in peripheral nerves. In this study, we certainly demonstrated that the association between ST and TP modulated the aging of the nerve components, such that practically all parameters analyzed remained similar to IC, thus retarding aging. The results demonstrated here are partially explained by the probable action of strength training on neurotrophic factors. However, TP association with ST may have potentiated this effect. Jia et al. (2016) analyzed a senescence model and clearly demonstrated that the T action on central plasticity is mediated by the ARs. Yang and Arnold (2000) demonstrated that the action of BDNF on AR is testosterone-dependent. Thus, it is possible that the training action on the adaptations of peripheral nerves is mediated by steroid hormones (Wood et al., 2012).

Bhasin et al. (2000) demonstrated that the association of strength training and anabolic steroid use is more effective in increasing muscle mass and strength than each therapy alone. It is known that testosterone acts via interaction with its respective receptor (AR), so strength training is able to increase AR mRNA expression (Willoughby and Taylor, 2004), enhancing the effects of its administration.

Thus, we conclude that: (1) management of TP is effective in stimulating settings in the morphology of the tibial nerve; (2) ST is a potent modulator of all morphometric parameters and an important tool to increase strength through progressive increases in relative volume of training; and (3) the combination of TP and ST appears no different than ST alone to induce changes in the morphology of tibial nerve.

## Author Contributions

WKN trained the animals, prepared and analyzed the material, wrote the final manuscript. WAS trained animals and prepared the material. APC analyzed the material and revised the final elaboration of the manuscript. RABN prepared and analyzed the material. CAA analyzed the material, read and revised the final manuscript text. EFG tutored the entire process.

## Conflict of Interest Statement

The authors declare that the research was conducted in the absence of any commercial or financial relationships that could be construed as a potential conflict of interest.
